# Breakfast skipping and cognitive and emotional engagement at school: a cross-sectional population-level study

**DOI:** 10.1017/S1368980021004870

**Published:** 2022-12

**Authors:** Hero Moller, Alanna Sincovich, Tess Gregory, Lisa Smithers

**Affiliations:** 1 Telethon Kids Institute, University of Western Australia, Level 7, 31 Flinders St., Adelaide, South Australia 5000, Australia; 2 School of Public Health, University of Adelaide, Level 5, Rundle Mall Plaza, Adelaide, South Australia 5000, Australia; 3 School of Health and Society, University of Wollongong, Wollongong, Australia; 4 Robinson Research Institute, Norwich Centre, North Adelaide, Australia

**Keywords:** Breakfast skipping, Breakfast consumption, Children and adolescents, Student engagement, School climate, Wellbeing and Engagement Collection

## Abstract

**Objective:**

Research on the consequences of breakfast skipping among students tends to focus on academic outcomes, rather than student well-being or engagement at school. This study investigated the association between breakfast skipping and cognitive and emotional aspects of school engagement.

**Design:**

Cross-sectional study using data from a population-level survey of children and adolescents’ well-being and engagement at school. Linear regression with adjustment for confounders was used to estimate the effect of breakfast skipping on school engagement.

**Setting:**

Government schools (i.e. public schools) in South Australia.

**Participants:**

The participants were students, Grades 4–12, who completed the Wellbeing and Engagement Collection in 2019. The analysis sample included 61 825 students.

**Results:**

Approximately 9·6 % of students reported always skipping breakfast, with 35·4 % sometimes skipping and 55·0 % never skipping. In the adjusted linear regression models, children and adolescents who always skipped breakfast reported lower levels of cognitive engagement (*β* = −0·26 (95 % CI −0·29, −0·25)), engagement with teachers (*β* = −0·17 (95 % CI −0·18, −0·15)) and school climate (*β* = −0·17 (95 % CI −0·19, −0·15)) compared with those who never skipped breakfast, after controlling for age, gender, health, sleep, sadness and worries, parental education, socio-economic status and geographical remoteness.

**Conclusion:**

Consistent with our hypothesis, skipping breakfast was associated with lower cognitive and emotional engagement, which could be due to mechanisms such as short-term energy supply and long-term health impacts. Therefore, decreasing the prevalence of breakfast skipping could have a positive impact on school engagement.

A healthy breakfast is an important part of the diets of children and adolescents. Healthy breakfasts are typically high in nutrients like calcium and fibre, and going without breakfast can become a missed opportunity for the necessary energy and nutrients for growth and healthy development^(1)^. Studies show that breakfast skipping is often clustered with other unhealthy behaviours, such as increased intake of discretionary food and low physical activity^(2–4)^. There is also evidence that skipping breakfast can lead to poorer academic outcomes, making research exploring the relationship between breakfast and school outcomes of particular interest to a range of stakeholders, including government departments, educators and public health researchers^(1,5)^.

An international systematic review of breakfast habits among children found that the prevalence of breakfast skipping ranged from 12 to 34 %^(1)^. In this review, breakfast skipping was more prevalent among females, older children and children from lower socio-economic backgrounds. The prevalence of breakfast skipping was also higher among adolescents who reported smoking, had low physical activity, dieted and had body weight concerns. The most common reasons reported for skipping breakfast were lack of time, no appetite or dieting to lose weight. Further, skipping breakfast for some children may be influenced by the family level and community factors, including food insecurity and family structure^(6–9)^. In Australia, the setting of this present study, the prevalence of breakfast skipping also tends to be higher among females and older children and adolescents^(10)^. The 2011–2012 National Nutrition and Physical Activity Survey (*n* 1592; 2–17 years old) found that 18·6 % of females and 13·2 % of males had skipped breakfast on at least one of two recall days, while the prevalence of skipping on both recall days was 3·8 % among females and 1·4 % among males^(3)^.

Breakfast consumption has been associated with school performance and academic achievement^(5,11,12)^. An Australian study found that breakfast skipping among 8–9-year-old children was associated with poorer academic outcomes as reported by teachers 2 years later as well as literacy and numeracy outcomes^(11)^. A study in the Netherlands found that among students 11–18 years of age, skipping breakfast on any school day was associated with the poorer end of term grades^(5)^. A systematic review on the effect of breakfast consumption on cognitive outcomes in children and adolescents found that eating breakfast had a positive effect on certain aspects of cognitive function measured (e.g. attention, executive function, and memory) within 4 h post-breakfast, compared with skipping breakfast^(12)^. The authors also concluded that the effects of breakfast consumption were strongest among undernourished children, indicating that in some cases reducing the prevalence of breakfast skipping could have considerable positive effects among children most in need^(12)^. Many observational studies on the effects of breakfast on academic and psychosocial outcomes, however, have failed to account for potential confounding by socio-economic factors^(13–15)^.

In some countries, such as the USA, school breakfast programs have become a popular intervention to increase breakfast consumption. The evidence of school breakfast programs’ impact on academic performance and behaviour in schools remains mixed. Randomised controlled trials of school breakfast programs in the USA, UK and New Zealand found little impact on outcomes such as attention, concentration, memory, behaviour, school attendance or academic achievement^(16–18)^. On the other hand, some studies from the USA have found positive effects of breakfast programs on maths and reading scores and school attendance^(19,20)^. It should also be noted that many studies of school breakfast programs report low attendance and no decrease in the prevalence of breakfast skipping^(17,18,21,22)^.

The relationship between breakfast skipping and academic outcomes has been well studied, but the relationship between breakfast skipping and other school-related outcomes, such as school engagement, is relatively understudied^(5,11,12,23)^. School engagement is a multifaceted construct that includes three main dimensions: behavioural, emotional and cognitive engagement^(24)^. These dimensions are dynamic in themselves and capture how involved students are with school (behavioural), effort applied to learning (cognitive), attitudes to peers and teachers at school and how aspects of school are valued (emotional). School engagement has been identified as an important and potentially malleable predictor of positive school and later life outcomes^(25,26)^. In a longitudinal Australian study, school engagement was associated with continuing education post-school and higher status occupations, after adjusting for socio-economic status (SES) in childhood and school-level variables (e.g. government or private, number of students)^(27)^. Thus, school engagement may contribute to students’ well-being while they are still at school and reduce rates of school dropout, making it an important outcome to study in addition to academic performance^(28,29)^.

Using data from a population-level survey of children’s well-being and engagement at school, the current study aimed to investigate the association between breakfast skipping and school engagement. In light of the studies that have previously found associations between breakfast skipping and other school outcomes, we hypothesised that there would be an association between breakfast skipping and school engagement, with children who skip breakfast experiencing lower cognitive and emotional engagement at school. Furthermore, since the systematic review identified differences in the prevalence of breakfast skipping by age, sex and SES^(1)^, we investigated whether these factors also modified the association between breakfast skipping and school engagement, as this information may be important for targeting future breakfast policies and interventions.

## Methods

### Data source

The Wellbeing and Engagement Collection (WEC) is a population-level survey conducted annually in South Australian schools. The current study utilised data from the 2019 WEC. All South Australian schools (*n* 715) were invited to participate in the 2019 WEC, and school-level participation rates of 89 % (government/public schools), 52 % (Catholic schools) and 19 % (independent schools) were achieved. A total of 95 973 students, from Grade 4 to 12, completed the WEC in 2019. In the current study, only WEC data from the students in government/public schools was utilised because this could be linked to enrolment census data held by the South Australian Department for Education to provide information on a range of child and family-level socio-demographic confounders.

The WEC survey is designed to be completed over one to two class periods at school, taking approximately 25–45 min to complete. The WEC measures four domains of student well-being and engagement: Emotional Wellbeing, Engagement with School, Learning Readiness, and Health and Wellbeing out of school, and many different constructs within each domain. These constructs (e.g. happiness, sadness, optimism) are measured using a combination of multi-item scales and single items^(30)^. In this study, one measure of cognitive engagement and two measures of emotional engagement (Emotional Engagement with Teachers and School Climate) were used as outcomes. These are described in more detail below.

### Ethical approval and consent

All identifying information was removed from the dataset (i.e. name, address, date of birth) before receiving it for analysis to ensure confidentiality. The sampling method consisted of inviting all primary and secondary schools in South Australia to participate. Schools were then free to decide if all or some classes would complete the WEC, and the parents and guardians of children in these classes received an information letter. This gave them time to withdraw their child if they so wished, and children could opt out themselves before or during the survey at any time.

### Participants

**Fig. 1:**
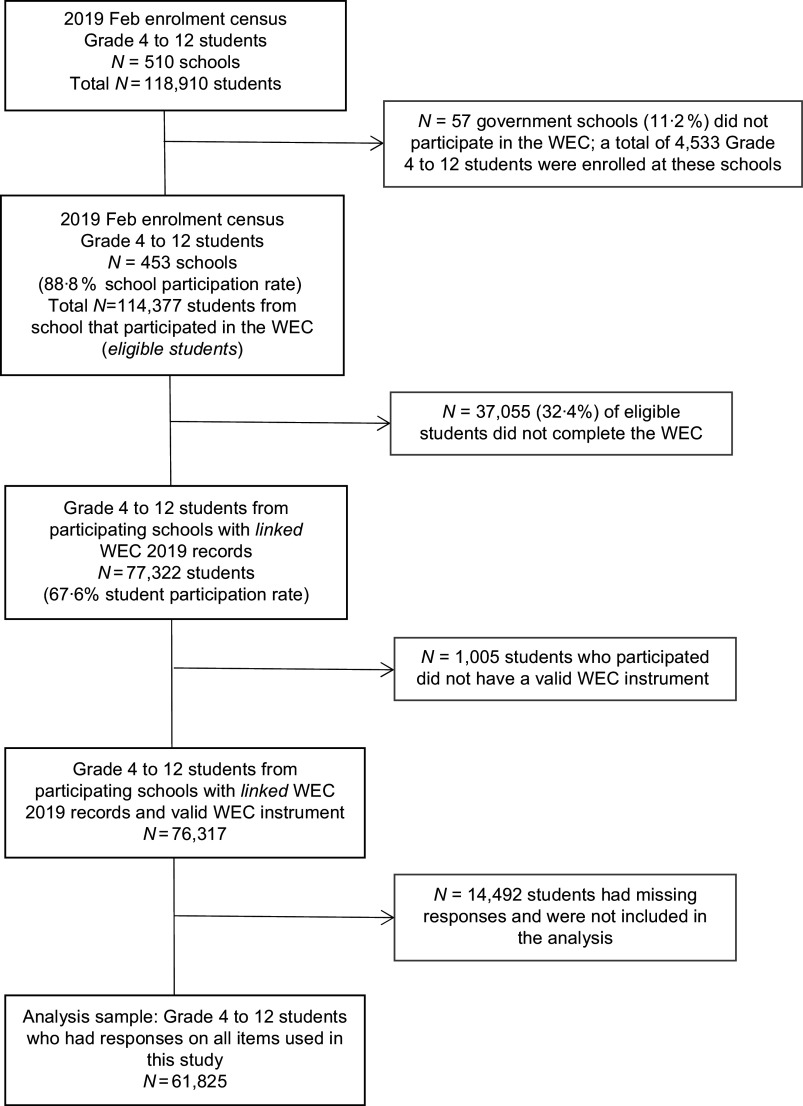
Flow chart of participants/sample

The participants were students from government schools in South Australia, Grades 4–12, who completed the WEC in 2019. Figure 1 shows the number of schools and students in the 2019 enrolment census (*n* 118 910). A total of 453 government schools participated in the WEC, and 77 322 students from these schools completed the WEC survey (67·6 % student participation rate). A small number of these students (*n* 1005, 1·3 %) started the survey but did not complete enough items such that their responses were deemed invalid. The response sample was 76 317 students, of which 14 492 students were excluded as they had missing data on one or more of the outcome, exposure or confounder variables used in the analysis (see online Supplemental Table 1 for characteristics of response sample). The analysis sample included 61 825 students who had observations on all items used in this study (see Fig. 1).

### Measures

#### Exposure

##### Breakfast skipping

Breakfast consumption was measured using a single item that asked students ‘how often do you eat breakfast?’ Students responded using an eight-point scale (Never, Once a week, 2 times a week, …, 6 times a week, Every day). This item was recoded into 1 = never skippers (eat breakfast every day), 2 = sometimes skippers (eat breakfast 1 to 6 times a week) and 3 = always skippers (never eats breakfast) to compare children who eat breakfast everyday with children who skip breakfast. Similar self-report measures of breakfast consumption have been used in previous studies^(31,32)^.

#### Outcomes

##### Cognitive engagement

The five-item cognitive engagement scale measures how students engage with learning and how they apply themselves. It includes items such as ‘I work hard on learning’, ‘When I found something hard I tried another way’ and ‘No matter who you are, you can change your intelligence’. Students responded using a five-point Likert scale (Never = 1 to Always = 5), and the mean of the five items was calculated. This item was adapted from the twelve-item cognitive engagement subscale, from the Teaching for Effective Learning School Engagement survey (created by the SA Department for Education), for the WEC to expand its measures of student engagement^(30)^.

##### Emotional engagement with teachers

The five-item emotional engagement with teachers scale includes items such as ‘I get along well with most of my teachers’, ‘Most of my teachers are interested in my wellbeing’ and ‘If I need extra help, I will receive it from my teachers’. Students responded using a four-point Likert scale (Strongly Disagree = 1 to Strongly Agree = 4), and the mean of the five items was calculated. It was originally used in the Programme for International Student Assessment (PISA) Student Context Questionnaire, which was developed by Organisation for Economic Cooperation and Development (OECD) and has been used across OECD countries since 2000^(33)^.

##### School climate

The three-item school climate scale includes the items: ‘Teachers and students treat each other with respect in this school’, ‘People care about each other in this school’ and ‘Students in this school help each other, even if they are not friends’. Students responded using a five-point Likert scale (Strongly Disagree = 1 to Strongly Agree = 5), and the mean of the three items was calculated. Originally adapted from the Self-Beliefs/Academic Self-efficacy scale, this item was used in this study to capture emotional engagement to the school more broadly^(34,35)^.

All three of these scales have been psychometrically tested, showing high internal reliability among children in all grades with Cronbach’s *α* statistics ranging from 0 80 to 0 89^(30)^.

#### Confounding

Potential confounders of the relationship between breakfast skipping and school engagement were selected prior to the analysis based on a causal model using directed acyclic graphs and consulting the literature (see online Supplemental Fig. 1). These included age, gender, overall health, sadness and worry; highest level of parent education; community level SES and geographical remoteness. Overall health was based on the item ‘In general, how would you describe your health?’ with a four-point response scale (poor = 1 to excellent = 4) categorised into low (poor and fair), medium (good) and high (excellent). Sleep was measured using a single item that asked students ‘How often do you get a good night’s sleep?’, with response options ‘0 = Never’ through to ‘7 = Everyday’. Sadness was measured using a three-item scale, which was adapted from the Depression subscale in the Seattle Personality Questionnaire^(34,36)^. The sadness scale includes items such as ‘I feel unhappy a lot of the time’ and a five-point Likert scale (Strongly Disagree = 1 to Strongly Agree = 5). The three-item worry/anxiety scale was used to measure students’ worries, including items such as ‘I worry about things at home’ and a five-point Likert scale (Strongly Disagree = 1 to Strongly Agree = 5). The following variables were made available through the linked school enrolment census data, which is based on the information given by parents or caregivers. Parent education was based on the highest level of education attained by a student’s parents/caregivers (i.e. year 9 or equivalent or below; year 10 or equivalent; year 11 or equivalent; year 12 or equivalent; Certificate I to IV; Advanced Diploma or Diploma or Bachelor Degree or above). SES was based on the 2016 Socio-Economic Indexes for Areas’ Index of Relative Socio-economic Advantage and Disadvantage for students’ postcodes, which compares areas according to different aspects of socio-economic disadvantage or advantage. The Accessibility and Remoteness Index of Australia, according to students’ residential postcodes (i.e. zipcodes), was used to distinguish students who live in major cities, inner regional, outer regional, remote or very remote areas of South Australia. The remote and very remote categories were combined due to small numbers. The constructs and measures used in this study are measured the same way (i.e. using the same items/scales) across all age groups.

### Analysis

A series of linear regression models were used to test the effect of breakfast skipping on cognitive and emotional engagement before and after adjustment for confounders (described above). The children and adolescents who never skip breakfast were the reference category for the exposure variables in all analyses because most children were in this category and eating breakfast everyday is considered to be the ideal frequency. All three school engagement outcomes were confirmed to be normally distributed prior to analysis. A small number of students did not have data on all three outcomes. We explored if the effect estimates differed according to whether the analysis included students only with all three outcomes, exposures and confounders (main analysis; complete case sample), from an analysis where students had valid scores on one specific outcome but missing scores on another outcomes (response sample; see online Supplemental Table 2). The sample size for these three analyses varied between 63 441 and 64 001, depending on the amount of missing data on each outcome. The effect estimates using the complete case sample and the response sample were the same, suggesting non-completion on some outcomes did not influence the results. The effect measure modification analyses (see online Supplemental Appendix A) were conducted according to best epidemiological practice^(37)^. The relative excess risk due to interaction was calculated to estimate the extent of effect measure modification on the risk difference scale, as this is considered most relevant for public health. The exposure, outcomes and effect modification variables were dichotomised for these analyses (see online Supplemental Appendix A).

Analyses were conducted using Stata se version 16.

## Results

Table 1 describes the demographic characteristics of students included in the analysis according to breakfast skipping categories (i.e. never, sometimes or always skips breakfast). In the analysis sample, 55·0 % of students reported that they ate breakfast everyday, 35·4 % reported sometimes skipping breakfast and 9·6 % reported always skipping breakfast (Table 1). Always skippers tended to be older and female, with lower parent education and a higher proportion lived in lower socio-economic areas and regional areas. Children who reported always skipping breakfast often reported less nights of good sleep and increased sadness and worry compared with sometimes and never skippers. Of note is that the proportion reporting high overall health among never skippers (41·4 %) was similar to the proportion reporting low overall health among always skippers (48·5 %). All outcome variables showed a gradient across never, sometimes and always skipping, with students that never skip breakfast reporting higher levels of cognitive engagement, engagement with teachers and school climate. Students that never skip breakfast reported mean (sd) scores for cognitive engagement, emotional engagement and school climate of 4·0 (0·8), 3·2 (0·5) and 3·7 (0·8), respectively, while children who always skip breakfast reported scores of 3·2 (1·0), 2·8 (0·7) and 3·0 (1·0). Mean scores of students that sometimes skip breakfast were always in the middle. The demographic characteristics of the analysis sample were similar to the response sample (see online Supplemental Table 1).

**Table 1:** Characteristics of analysis sample according to exposure of breakfast skipping (*n* 61 825)

Exposure:	Never skips (*n* 34 018)			Sometimes skips (*n* 21 873)			Always skips (*n* 5934)			Total (*n* 61 825)		
	*n*	% or Mean	sd	*n*	% or Mean	sd	*n*	% or Mean	sd	*n*	% or Mean	sd
Outcomes												
Cognitive engagement	38 313	4·0	0·8	24 819	3·6	0·8	6671	3·2	1·0	61 825	3·8	0·8
Emotional engagement with teachers	38 008	3·2	0·5	24 602	3·0	0·5	6642	2·8	0·7	61 825	3·1	0·6
School climate	38 082	3·7	0·8	24 662	3·3	0·8	6655	3·0	1·0	61 825	3·5	0·9
Confounders												
Age (years)	34 018	12·4	2·5	21 873	13·5	2·5	5934	14·3	2·6	61 825	12·8	2·6
Gender												
Male	18 396	54·1		9956	45·5		2461	41·5		30 813	49·8	
Female	15 420	45·3		11 714	53·6		3357	56·6		30 491	49·3	
Other	202	0·6		203	0·9		116	1·9		521	0·8	
Overall health												
High	14 087	41·4		4414	20·2		815	13·7		19 316	31·2	
Medium	15 575	45·8		11 058	50·6		2244	37·8		28 877	46·7	
Low	4356	12·8		6401	29·3		2875	48·5		13 632	22·1	
Sleep*	34 018	5·1	2·0	21 873	3·7	2·2	5934	2·5	2·4	61 825	4·4	2·3
Sadness	34 018	2·5	1·0	21 873	3·0	1·0	5934	3·3	1·0	61 825	2·8	1·0
Worry	34 018	2·9	1·1	21 873	3·3	1·0	5934	3·5	1·1	61 825	3·1	1·1
Highest parent education												
Year 9 or equivalent or below	636	1·9		475	2·2		147	2·5		1258	2·0	
Year 10 or equivalent	1006	3·0		985	4·5		362	6·1		2353	3·8	
Year 11 or equivalent	1771	5·2		1605	7·3		668	11·3		4044	6·5	
Year 12 or equivalent	3587	10·5		2822	12·9		882	14·9		7291	11·8	
Certificate I–IV	8929	26·3		6730	30·8		1941	32·7		17 600	28·5	
Advanced diploma or diploma	4858	14·3		3226	14·8		836	14·1		8920	14·4	
Bachelor degree or above	13 231	38·9		6030	27·6		1098	18·5		20 359	32·9	
Socio-economic status†												
Most disadvantaged 1	7102	20·9		5821	26·6		1996	33·6		14 919	24·1	
2	5264	15·5		3846	17·6		1080	18·2		10 190	16·5	
3	5456	16·0		3610	16·5		941	15·9		10 007	16·2	
4	8719	22·6		4400	20·1		1060	17·9		13 134	21·2	
Most advantaged 5	9474	25·0		4196	19·2		857	14·4		13 575	22·0	
Geographical remoteness‡												
Major cities	24 524	72·1		14 922	68·2		3980	67·1		43 426	70·2	
Inner regional	4728	13·9		3453	15·8		989	16·7		9170	14·8	
Outer regional	3700	10·9		2732	12·5		762	12·8		7194	11·6	
Remote/very remote	1066	3·1		766	3·5		203	3·4		2035	3·3	

* Sleep measures how many nights, on average, do students feel they get a good night’s sleep (0–7).

† Socio-Economic Indexes for Areas Index of Relative Socio-economic Advantage and Disadvantage is a set of measures derived from Australian Bureau of Statistics census information that summarise different aspects of socio-economic conditions in an area.

‡ Accessibility and Remoteness Index of Australia (i.e. geographical remoteness).

The adjusted and unadjusted results of the linear regression of the association between breakfast skipping and school engagement outcomes are shown in Table 2. The results are presented as unstandardised regression coefficients (i.e. the mean differences in outcome(s) relative to the reference group of never breakfast skippers) and 95 % CI. In the adjusted models, children who always skipped breakfast reported lower levels of cognitive engagement (*β* = −0·26 (95 % CI −0·29, −0·25), *P* < 0·0001), emotional engagement with teachers (*β* = −0·17 (95 % CI −0·18, −0·15), *P* < 0·0001) and school climate (*β* = −0·17 (95 % CI −0·19, −0·15), *P* < 0·0001), compared with those who never skipped breakfast. Children who sometimes skipped breakfast also reported lower levels of engagement compared with children who never skipped breakfast, but not as low as always skippers. For example, after adjustment, the effect of always skipping on cognitive engagement was −0·26 (95 % CI −0·29, −0·25), *P* < 0·0001)), but the effect of sometimes skipping was only −0·08 (95 % CI −0·09, −0·07), *P* < 0·0001)).

**Table 2:** Linear regression results for the effect of skipping breakfast on school engagement (*n* 61 825)

	Cognitive engagement				Emotional engagement with teachers				School climate			
	Unadjusted *β*	95 % CI	Adjusted *β*	95 % CI	Unadjusted *β*	95 % CI	Adjusted *β*	95 % CI	Unadjusted *β*	95 % CI	Adjusted *β*	95 % CI
Never skips breakfast	Ref		Ref		Ref		Ref		Ref		ref	
Sometimes skips breakfast	−0·35	−0·37, −0·34	−0·08	−0·09, −0·07	−0·22	−0·23, −0·21	−0·06	−0·07, −0·05	−0·32	−0·33, −0·30	−0·05	−0·06, −0·03
Always skips breakfast	−0·76	−0·78, −0·74	−0·26	−0·29, −0·25	−0·44	−0·46, −0·43	−0·17	−0·18, −0·15	−0·65	−0·68, −0·63	−0·17	−0·19, −0·15

Adjusted for: age, gender, overall health, sleep, sadness and worries scales, highest level of parent education, student Socio-Economic Indexes for Areas Index of Relative Socio-economic Advantage and Disadvantage and student Accessibility and Remoteness Index of Australia.

*β* is unstandardised beta-coefficient.

Across all school engagement outcomes, we found limited evidence of effect measure modification by sex, socio-economic position or age (see online Supplemental Tables 3–6). For example, the within-stratum effects suggest a 19 % higher risk of poor cognitive engagement among males who sometimes/always skip breakfast compared with those who never skip breakfast (RR 1·19 (95 % CI 1·12, 1·26)) and a 33 % higher risk among females (RR 1·33 (95 % CI 1·24, 1·43)). However, the relative excess risk due to interaction of 0·01 (95 % CI −0·09, 0·12) indicates no effect measure modification by sex on the risk difference scale. That is, the combined risks of both breakfast skipping and sex was not greater than the sum of the individual risks of breakfast skipping and sex. There was limited evidence of effect measure modification by socio-economic position or age, with five of the six relative excess risk due to interaction estimates close to zero with 95 % CI that included zero. The single exception was the effect of breakfast skipping on school climate by age, with results suggesting this effect was larger in primary school compared with high school students. Full results for the effect measure modification analyses can be found in Supplemental Appendix A.

## Discussion

There has been a great deal of research on the association between breakfast consumption and school outcomes, but these studies tend to focus on academic achievement and cognitive function (e.g. memory tests) rather than how students engage with schooling and teachers. This current study explored the latter, focusing on the relationship between breakfast skipping and two dimensions of school engagement; cognitive and emotional engagement. Consistent with our hypothesis, we found that skipping breakfast was associated with lower cognitive and emotional engagement at school. Children who reported always skipping breakfast had lower engagement scores compared with those that sometimes skipped breakfast or never skipped breakfast. These findings build on prior observational research of breakfast and school outcomes that used small sample sizes (ranging from *n* 97 to *n* 294) and/or did not adjust for potential confounding^(5,13–15,23)^. For instance, one study found that regular breakfast consumption was associated with an improved emotional state among adolescents in South Korea (*n* 62 276), but had not adjusted for any confounding^(13)^. Although the prevalence of breakfast skipping is reported to differ according to age, sex and socio-economic position^(1)^, we found limited evidence that this extended to the association between skipping and most outcome measurements of cognitive and school engagement. The only exception was the association between breakfast skipping and school climate, where the effect of breakfast skipping on school climate appeared larger for a primary school than high school students.

Considering that research has shown there is a substantial decline in school engagement as students move from primary school to high school^(24,38)^, comparing the effect of this transition on school engagement with the effects observed in our study might offer a helpful comparison to put the magnitude of these effects in perspective^(39)^. Normative data from the WEC suggests that the difference in mean scores observed for each outcome between children in their last 2 years of primary school compared with their first 2 years of high school in the 2019 WEC were −0·23 for cognitive engagement, −0·19 for emotional engagement with teachers and −0·27 for school climate^(30)^. This suggests the adjusted effect sizes observed, for cognitive engagement (−0·26, (95 % CI −0·29, −0·24)) and emotional engagement with teachers (−0·17, (95 % CI −0·18, −0·15)) especially, are similar in magnitude to the decrease in engagement already experienced by students as they move through school, which is substantial.

These findings are consistent with previous research on the association between breakfast and school outcomes, such as academic achievement, which often require high levels of cognitive engagement to succeed^(5,40,41)^. A study in the UK (*n* 294, 14–15 years) found that after controlling for age, sex, BMI, ethnicity and SES, students who rarely (never or once a week) consumed breakfast on school days scored lower General Certificate of Secondary Education grades^(23)^. In an Australian study, 8–9-year-olds who skipped breakfast (*n* 2280) had poorer teacher-reported academic outcomes than children who did not skip breakfast. However, because there was only a slight difference observed for objective National Assessment Program – Literacy and Numeracy (NAPLAN) results, the authors argued the results might be due to unmeasured cofounding between children who regularly skipped breakfast and teacher-reported outcomes^(11)^. Similar to our study, these findings both suggest there is something about eating breakfast that can indeed impact school outcomes. Furthermore, levels of school engagement might be an indirect pathway through which breakfast impacts academic outcomes^(5)^.

One possible mechanism for how breakfast influences school outcomes is that eating breakfast promotes glucose uptake in the brain. Glucose is the brain’s main fuel source and provides energy to concentrate at school^(42)^. Hence, if skipping breakfast means that children arrive at school hungry, are distracted and do not have much energy, they may be less emotionally and cognitively engaged^(5)^. The quality of the breakfast may be pertinent if skipping breakfast was compared with eating a healthy breakfast. For instance, evidence shows low glycaemic index foods sustain blood glucose levels for longer periods, which can improve attention at school, compared with fasting or eating a high glycaemic index breakfast^(43)^. Over a longer period of time, through improving the overall quality of children’s diets, breakfast could impact school outcomes through long-term overall health promotion, which itself has been found to be associated with school engagement^(44)^. A combination of these mechanisms could have contributed to the effects observed in this study. Increased breakfast consumption has been shown to have a stronger impact on school outcomes among undernourished children or children from lower socio-economic backgrounds, suggesting where gains can be made (i.e. in nutritional quality or less discretionary foods) it could have an impact on how students engage with school^(12,19,45)^.

Strengths of this study were adjusting for a range of confounders and the large population-based sample, which allowed for the detection of small differences in engagement levels. There are some limitations that should be considered. While we adjusted for a community-level measure of SES and parental education, it is possible that the effect estimates remain residually confounded through factors such as socioeconomically patterned attitudes to health and education or family structure^(8,46,47)^. Some measurement bias (e.g. low content validity) is common when using large population-level surveys, which can lead to unmeasured confounding. Research on the 2019 WEC has shown there is some bias in the WEC sample with children from more socio-economically disadvantaged backgrounds under-represented^(48)^. Further, there was no information on why children skipped breakfast. This information may provide a clearer relationship between breakfast skipping and school engagement, especially if the reason represents a confounding relationship, such as food insecurity. On the other hand, if most breakfast skippers reported it is because they are not hungry in the mornings, appetite is less likely to be a direct influence on school engagement.

The possibility that children report skipping breakfast because of food insecurity has driven much of the concern over breakfast skipping, however it is rarely given as a reason for skipping breakfast^(49)^. Reasons that are commonly given by adolescents for skipping breakfast include lack of time, lack of appetite and/or dieting, and attitudes towards breakfast tend to be the strongest predictor of breakfast consumption^(50–52)^. While interventions (e.g. school breakfast programs) aiming to increase breakfast consumption rarely achieve their goal, the most successful among them include persuasive messaging that increases positive attitudes towards breakfast^(22,53,54)^. As such, schools and education departments may want to explore the impact of low cost, health promotion interventions in classrooms focussed on attitudes towards breakfast on breakfast consumption and school outcomes, including levels of engagement.

To conclude, this study demonstrated an association between skipping breakfast and cognitive and emotional engagement at school, among a large sample of school students in Australia, after adjustment for a comprehensive set of child and family-level confounders. Similar to studies on the effects of breakfast on academic performance, our study shows there could also be an important link between breakfast and students’ engagement. Short-term energy supply and long-term health impacts are two possible mechanisms that may explain this association. Considering these findings, decreasing the prevalence of breakfast skipping could have a positive impact on school engagement.

## Supplementary material

For supplementary material accompanying this paper visit https://doi.org/10.1017/S1368980021004870. click here to view supplementary material
