# Supplementary material for: Breakfast skipping and cognitive and emotional engagement at school: a cross-sectional population-level study
Source: Public Health Nutr. 2021 Dec 16;25(12):3356–65. doi: 10.1017/S1368980021004870 (PMC9991782; doi:10.1017/S1368980021004870)
Supplement: Supplementary file 1 [file S1368980021004870sup001.docx]

**Supplementary Materials**

**Supplementary Figure 1. Directed Acyclic Graph of the effect of breakfast skipping on cognitive and emotional engagement**


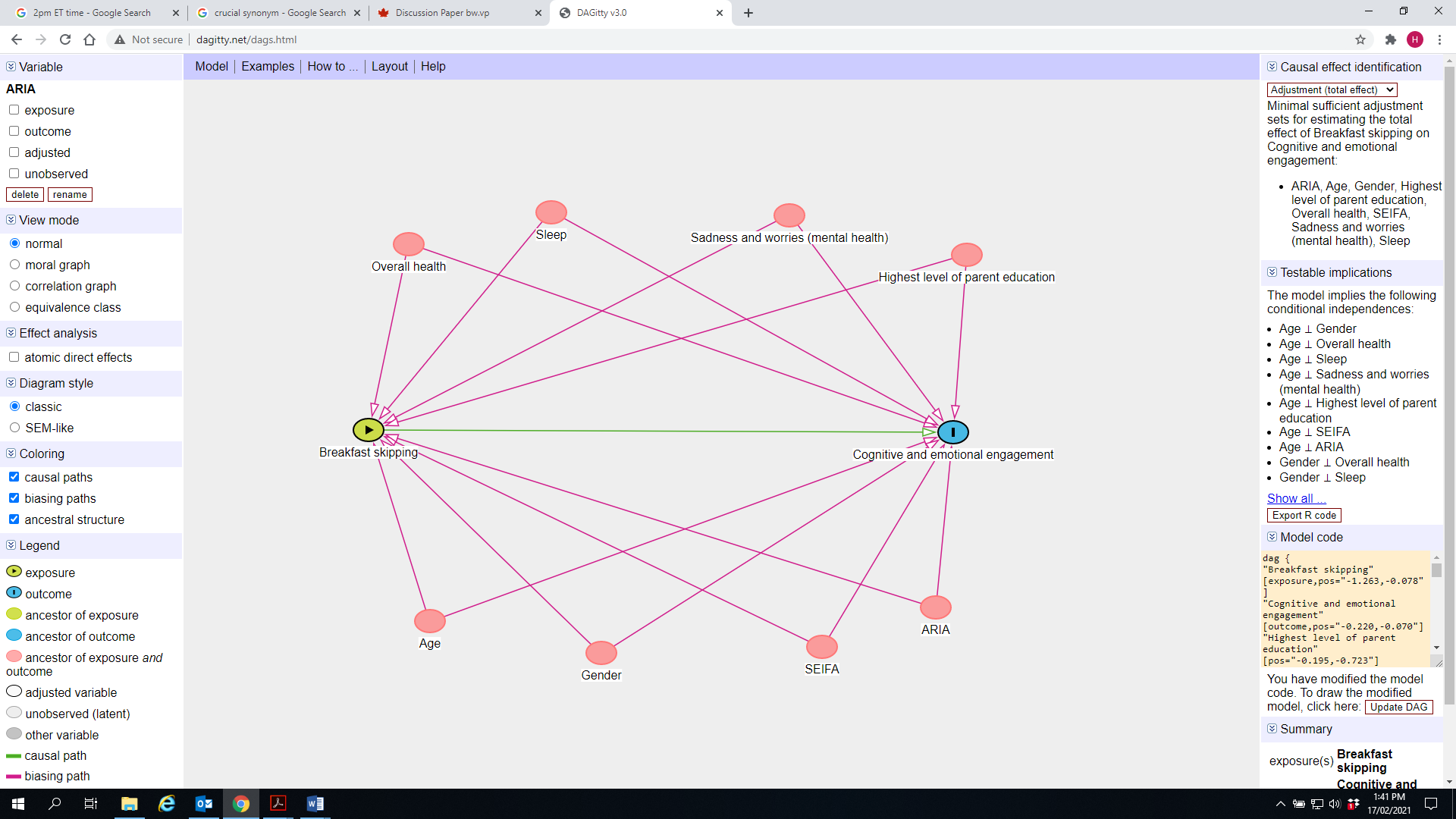


Made using <http://www.dagitty.net/dags.html>

**Supplementary Table 1. Characteristics of response and analysis samples**

|  | Response sample  n=76,317 | | Analysis sample  n=61,825 | |
| --- | --- | --- | --- | --- |
|  | N | % or M (SD) | N | % or M (SD) |
| Outcomes |  |  |  |  |
| Cognitive engagement | 71,728 | 3.8 (0.8) | 61,825 | 3.8 (0.8) |
| Missing | 4,589 | - | - | - |
| Emotional engagement with teachers | 71,797 | 3.1 (0.6) | 61,825 | 3.1 (0.6) |
| Missing | 4,520 |  |  |  |
| School climate | 71,917 | 3.5 (0.9) | 61,825 | 3.5 (0.9) |
| Missing | 4,400 | - | - | - |
| Exposure |  |  |  |  |
| Breakfast skipping |  |  |  |  |
| Never skips | 39,585 | 51.9 | 34,018 | 55.0 |
| Sometimes skips | 25,513 | 33.4 | 21,873 | 35.4 |
| Always skips | 6,885 | 9.0 | 5,934 | 9.6 |
| Missing | 4,334 | 5.7 | - | - |
| Confounders |  |  |  |  |
| Age (years) | 76,317 | 12.8 (2.6) | 61,825 | 12.9 (2.6) |
| Gender |  |  |  |  |
| Male | 38,512 | 50.5 | 30,813 | 49.8 |
| Female | 37,183 | 48.7 | 30,491 | 49.3 |
| Other | 622 | 0.9 | 521 | 0.8 |
| Overall Health |  |  |  |  |
| High | 22,096 | 29.0 | 19,316 | 31.2 |
| Medium | 32,811 | 43.0 | 28,877 | 46.7 |
| Low | 15,291 | 20.0 | 13,632 | 22.1 |
| Missing | 6,119 | 8.0 | - | - |
| Sleep^a^ | 71,329 | 4.4 (2.3) | 61,825 | 4.4 (2.3) |
| Missing | 4,988 | - | - | - |
| Sadness | 73,752 | 2.8 (1.0) | 61,825 | 2.8 (1.0) |
| Missing | 2,565 | - | - | - |
| Worry | 73,645 | 3.1 (1.1) | 61,825 | 3.1 (1.1) |
| Missing | 2,672 | - | - | - |
| Highest Parent Education |  |  |  |  |
| Year 9 or equivalent or below | 1,634 | 2.1 | 1,258 | 2.0 |
| Year 10 or equivalent | 3,017 | 4.0 | 2,353 | 3.8 |
| Year 11 or equivalent | 5,043 | 6.6 | 4,044 | 6.5 |
| Year 12 or equivalent | 8,753 | 11.5 | 7,291 | 11.8 |
| Certificate I to IV | 21,306 | 27.9 | 17,600 | 28.5 |
| Advanced Diploma or Diploma | 10,625 | 13.9 | 8,920 | 14.4 |
| Bachelor Degree or above | 23,594 | 30.9 | 20,359 | 32.9 |
| Missing | 2,345 | 3.1 | - | - |
| Socio-economic status^b^ |  |  |  |  |
| Most disadvantaged 1 | 19,952 | 26.1 | 14,919 | 24.1 |
| 2 | 12,603 | 16.5 | 10,190 | 16.5 |
| 3 | 11,986 | 15.7 | 10,007 | 16.2 |
| 4 | 15,660 | 20.5 | 13,134 | 21.2 |
| Most advantaged 5 | 15,815 | 20.8 | 13,575 | 22.0 |
| Missing | 301 | 0.4 | - | - |
| Geographical remoteness^c^ |  |  |  |  |
| Major Cities | 52,910 | 69.3 | 43,426 | 70.2 |
| Inner Regional | 11,105 | 14.6 | 9,170 | 14.8 |
| Outer Regional | 9,379 | 12.3 | 7,194 | 11.6 |
| Remote/Very Remote | 2,627 | 3.5 | 2,035 | 3.3 |
| Missing | 296 | 0.4 | - | - |

^a^ Sleep measures how many nights, on average, do students feel they get a good night’s sleep (0-7). ^b^ SEIFA IRSAD is a set of measures derived from ABS census information that summarise different aspects of socioeconomic conditions in an area. ^c^ Accessibility and Remoteness Index of Australia (ARIA; i.e. geographical remoteness)

**Supplementary Table 2. Linear regression results for the effect of skipping breakfast on school engagement using response sample**

|  | Cognitive engagement  n = 64,001 | | Emotional engagement with teachers  n = 63,451 | | School climate  n = 63,590 | |
| --- | --- | --- | --- | --- | --- | --- |
|  | Unadjusted  β (95% CI) | Adjusted  β (95% CI) | Unadjusted  β (95% CI) | Adjusted  β (95% CI) | Unadjusted  β (95% CI) | Adjusted  β (95% CI) |
| Never skips breakfast (ref) |  |  |  |  |  |  |
| Sometimes skips breakfast | -0.35 (-0.37, -0.34) | -0.08 (-0.09, -0.07) | -0.22 (-0.23, -0.21) | -0.06 (-0.07, -0.05) | -0.32 (-0.33, -0.30) | -0.05 (-0.06, -0.03) |
| Always skips breakfast | -0.76 (-0.78, -0.74) | -0.26 (-0.29, -0.24) | -0.44 (-0.46, -0.43) | -0.17 (-0.18, -0.15) | -0.65 (-0.68, -0.63) | -0.17 (-0.19, -0.16) |

Adjusted for: age, gender, overall health, sleep, sadness and worries scales, highest level of parent education, and student SEIFA IRSAD and ARIA. β is unstandardised beta-coefficient.

**Supplementary Appendix A. Effect-Measure Modification**

Our paper investigated the effect of breakfast skipping on school engagement. It was theorised that there may be differences in the relationship between breakfast skipping and school engagement among children with different socio-demographic characteristics. As such, we also conducted an investigation into whether there was effect modification by sex, socioeconomic position (SEP), and age.

The effect-measure modification analyses were conducted according to best epidemiological practice^1,2^. The relative excess risk due to interaction (RERI) was calculated to estimate the extent of effect-measure modification on the risk-difference scale, as this is considered most relevant for public health. A RERI > 0 indicates the effect-measure modification is positive (the effect of the exposure and the effect modifier operating together is greater than the effect of each added together). A RERI < 0 indicates the effect-measure modification is negative, and a RERI of 0 indicates there is no effect-measure modification on the risk-difference scale.

Cognitive engagement, school climate, and emotional engagement with teachers were used as continuous variables in the regressions but we used dichotomized outcomes when analysing effect-measure modification. These variables were dichotomised at <1 SD below the mean (‘low engagement’) and ≥1 SD below the mean (‘normal to high engagement’). The exposure was dichotomised into ‘always skips breakfast’ versus ‘sometimes or never skips breakfast’. We used primary school versus high school as a proxy for age, as this seemed the most logical demarcation for age-related change at the transition from primary to secondary school. For SEP, student Socio-Economic Indexes for Areas’ Index of Relative Socio-economic Advantage and Disadvantage (SEIFA IRSAD) quintiles were dichotomised into quintile 1 (‘least advantaged’) and quintiles 2-5 (‘more advantaged’). We used the same confounders that had been used in the regression models, but minus the potential effect-modifier.

Table S1 shows the interaction terms for the effect-measure modifier and exposure. However, as this is an insufficient approach for modelling effect-measure modification1 we present a full effect-measure modification analysis in Tables S2-S4. Across all school engagement outcomes, we found limited evidence of effect-measure modification by sex, SEP or age. For example, Table S2 shows the effect-measure modification of the association between breakfast skipping and cognitive engagement by sex. The RERI of 0.01 (95% CI -0.09, 0.12) indicates no effect-measure modification by sex on the risk-difference scale, i.e. the combined risks of both breakfast skipping and sex was not greater than the sum of the individual risks of breakfast skipping and sex. The within-stratum effects suggest a 19% higher risk of poor cognitive engagement among males who sometimes/always skip breakfast compared to those who never skip breakfast (RR 1.19 (95% CI 1.12, 1.26)) and a 33% higher risk among females (RR 1.33 (95% CI 1.24, 1.43)). Reflecting on these point estimates, and the fact that the CIs for the two RRs overlap, the 12% difference in magnitude between the sexes is trivial and would not warrant introducing sex-specific responses in school breakfast policies. Similarly, there was limited evidence of effect-measure modification by SEP or age, with five of the six RERI estimates close to zero with 95% CIs that included zero. The single exception was the effect of breakfast skipping on school climate by age, where the within-stratum effects suggest a 5% higher risk of poor school climate among high school students who sometimes/always skip breakfast compared to those who never skip breakfast (RR 1.05 (95% CI 0.99,1.11)) and a 27% higher risk among primary school students (RR 1.27 (95% CI 1.17, 1.37)), suggesting the effect of breakfast skipping on school climate was larger in primary school compared to high school students.

**Supplementary Table 3. Interaction effects of the effect of skipping breakfast on cognitive engagement, school climate, and emotional engagement with teachers by sex, SEP and student grade**

|  |  | Cognitive engagement | School climate | Emotional engagement with teachers |
| --- | --- | --- | --- | --- |
|  |  | β (95% CI) | β (95% CI) | β (95% CI) |
| Model 1  (n = 61,304) | Breakfast skipping x sex | 0.13 (0.02, 0.24) | 0.10 (-0.01,0.21) | 0.14 (0.04, 0.25) |
| Model 2  (n = 61,825) | Breakfast skipping x SEP | -0.01 (-0.13, 0.11) | -0.07 (-0.18, 0.05) | 0.02 (-0.10, 0.14) |
| Model 3  (n = 61,825) | Breakfast skipping x student grade | -0.05 (-0.16,0.06) | -0.31 (-0.41,-0.20) | -0.07 (-0.18,0.04) |

Notes. Model 1 adjusted for: age, overall health, sleep, sadness and worries scales, highest level of parent education, student Socio-Economic Indexes for Areas’ Index of Relative Socio-economic Advantage and Disadvantage (SEIFA IRSAD), and student Accessibility and Remoteness Index of Australia (ARIA). A small number of students selected “other” gender (n = 521). These cases were excluded from this analysis, so the sample is smaller than original analysis sample (n=61,825).

Model 2 adjusted for: age, gender, overall health, sleep, sadness and worries scales, highest level of parent education, and student ARIA.

Model 3 adjusted for: gender, overall health, sleep, sadness and worries scales, highest level of parent education, student SEIFA IRSAD and student ARIA.

**Supplementary Table 4. Effect-measure modification of the effect of breakfast skipping on cognitive engagement, school climate, and emotional engagement with teachers by sex (analysis sample, n=61,304)**

| **Cognitive engagement:** RERI **= 0.01** (95% CI -0.09, 0.12) | | | | | | | |
| --- | --- | --- | --- | --- | --- | --- | --- |
|  | Never skips | | | Sometimes/always skips | | |  |
|  | N Low engagement/  High engagement | | RR (95% CI) | N Low engagement/  High engagement | | RR (95% CI) | RR (95% CI) for low cognitive engagement within strata of sex |
| Male | 1,707/16,689 | | 1.00 (ref) | 2,537/9,880 | | 1.17 (1.11, 1.24) | 1.19 (1.12, 1.26) |
| Female | 969/14,451 | | 0.66 (0.62, 0.71) | 2,744/12,327 | | 0.91 (0.86, 0.96) | 1.33 (1.24, 1.43) |
| **School Climate:** RERI = **0.05** (95% CI -0.04, 0.15) | | | | | | | |
|  | | Never skips | | | Sometimes/always skips | |  |
|  |  | N Low engagement/  High engagement | RR (95% CI) | | N Low engagement/  High engagement | RR (95% CI) | RR (95% CI) for low school climate within strata of sex |
| Male | | 1,784/16,612 | 1.00 (ref) | | 2,210/10,207 | 1.10 (1.04, 1.17) | 1.10 (1.03, 1.17) |
| Female | | 1,161/14,259 | 0.74 (0.70, 0.80) | | 2,655/12,416 | 0.91 (0.86, 0.96) | 1.21 (1.13, 1.30) |
| **Emotional engagement with teachers:** RERI **= -0.02** (95% CI -0.12, 0.08) | | | | | | | |
|  | | Never skips | | | Sometimes/always skips | |  |
|  |  | N Low engagement/  High engagement | RR (95% CI) | | N Low engagement/  High engagement | RR (95% CI) | RR (95% CI) for low emotional engagement within strata of sex |
| Male | | 1,726/16,670 | 1.00 (ref) | | 2,413/10,004 | 1.25 (1.18, 1.33) | 1.28 (1.21, 1.36) |
| Female | | 923/14,397 | 0.62 (0.58, 0.67) | | 2,487/12,584 | 0.92 (0.87, 0.98) | 1.41 (1.31, 1.53) |

A small number of students selected “other” gender (n = 521). These cases were excluded from this analysis so the sample is smaller than original analysis sample (n=61,825). Relative excess risk due to interaction (RERI) and risk ratios (RR) are adjusted for: age, overall health, sleep, sadness and worries scales, highest level of parent education, student Socio-Economic Indexes for Areas’ Index of Relative Socio-economic Advantage and Disadvantage (SEIFA IRSAD), and student Accessibility and Remoteness Index of Australia (ARIA).

**Supplementary Table 5. Effect-measure modification of the effect of breakfast skipping on cognitive engagement, school climate, and emotional engagement with teachers by SEP (analysis sample, n=61,825)**

| **Cognitive engagement:** RERI **= -0.01** (95% CI -0.15, 0.12) | | | | | | | |
| --- | --- | --- | --- | --- | --- | --- | --- |
|  | Never skips | | | Sometimes/always skips | | |  |
|  | N Low engagement/  High engagement | | RR (95% CI) | N Low engagement/  High engagement | | RR (95% CI) | RR (95% CI) for low cognitive engagement within strata of SEP |
| More advantaged | 2,020/24,896 | | 1.00 (ref) | 3,698/16,292 | | 1.27 (1.20, 1.34) | 1.22 (1.16, 1.29) |
| Least advantaged | 694/6,408 | | 1.03 (0.95, 1.12) | 1,716/6,101 | | 1.26 (1.18, 1.34) | 1.33 (1.22, 1.44) |
| **School Climate:** RERI = **-0.06** (95% CI -0.19, 0.09) | | | | | | | |
|  | | Never skips | | | Sometimes/always skips | |  |
|  |  | N Low engagement/  High engagement | RR (95% CI) | | N Low engagement/  High engagement | RR (95% CI) | RR (95% CI) for low school climate within strata of SEP |
| More advantaged | | 2,207/24,709 | 1.00 (ref) | | 3,418/16,572 | 1.19 (1.13, 1.26) | 1.15 (1.09, 1.22) |
| Least advantaged | | 782/6,320 | 1.18 (1.09, 1.27) | | 1,577/6,240 | 1.28 (1.20, 1.37) | 1.16 (1.07, 1.27) |
| **Emotional engagement with teachers:** RERI **= -0.03** (95% CI -0.16, 0.10) | | | | | | | |
|  | | Never skips | | | Sometimes/always skips | |  |
|  |  | N Low engagement/  High engagement | RR (95% CI) | | N Low engagement/  High engagement | RR (95% CI) | RR (95% CI) for low emotional engagement within strata of SEP |
| More advantaged | | 2,093/24,823 | 1.00 (ref) | | 3,562/16,428 | 1.34 (1.27, 1.41) | 1.32 (1.25, 1.39) |
| Least advantaged | | 598/6,504 | 0.91 (0.84, 1.00) | | 1,472/6,345 | 1.23 (1.20, 1.32) | 1.41 (1.29, 1.55) |

RERI and RRs are adjusted for: age, gender, overall health, sleep, sadness and worries scales, highest level of parent education, and student ARIA.

**Supplementary Table 6. Effect-measure modification of the effect of breakfast skipping on cognitive engagement, school climate, and emotional engagement with teachers by student grade (analysis sample, n=61,825)**

| **Cognitive engagement:** RERI **= 0.00** (95% CI -0.13, 0.14) | | | | | | | |
| --- | --- | --- | --- | --- | --- | --- | --- |
|  | Never skips | | | Sometimes/always skips | | |  |
|  | N Low engagement/  High engagement | | RR (95% CI) | N Low engagement/  High engagement | | RR (95% CI) | RR (95% CI) for low cognitive engagement within strata of grade |
| Primary school | 1,460/19,994 | | 1.00 (ref) | 1,684/9,293 | | 1.29 (1.21, 1.38) | 1.21 (1.13, 1.29) |
| High school | 1,254/11,310 | | 1.23 (1.15, 1.32) | 3,730/13,100 | | 1.46 (1.37, 1.55) | 1.25 (1.18, 1.33) |
| **School Climate:** RERI = **-0.34** (95% CI -0.53, -0.16) | | | | | | | |
|  | | Never skips | | | Sometimes/always skips | |  |
|  |  | N Low engagement/  High engagement | RR (95% CI) | | N Low engagement/  High engagement | RR (95% CI) | RR (95% CI) for low school climate within strata of grade |
| Primary school | | 1,301/20,153 | 1.00 (ref) | | 1,372/9,605 | 1.37 (1.28, 1.47) | 1.27 (1.17, 1.37) |
| High school | | 1,688/10,876 | 1.96 (1.83, 2.10) | | 3,623/13,207 | 1.96 (1.84, 2.09) | 1.05 (0.99, 1.11) |
| **Emotional engagement with teachers:** RERI **= 0.09** (95% CI -0.07, 0.24) | | | | | | | |
|  | | Never skips | | | Sometimes/always skips | |  |
|  |  | N Low engagement/  High engagement | RR (95% CI) | | N Low engagement/  High engagement | RR (95% CI) | RR (95% CI) for low emotional engagement within strata of grade |
| Primary school | | 1,360/20,094 | 1.00 (ref) | | 1,470/9,507 | 1.39 (1.29, 1.47) | 1.31 (1.23, 1.49) |
| High school | | 1,331/11,233 | 1.47 (1.37, 1.58) | | 3,564/13,266 | 1.83 (1.71, 1.95) | 1.29 (1.22, 1.38) |

RERI and RRs are adjusted for: gender, overall health, sleep, sadness and worries scales, highest level of parent education, student SEIFA IRSAD and student ARIA.

**References**

1. Knol, M. J., & VanderWeele, T. J. (2012). Recommendations for presenting analyses of effect modification and interaction. *International Journal of Epidemiology, 41*, 514-520.

2. VanderWeele, T. J., & M.J., Knol. (2014). A Tutorial on Interaction. *Epidemiologic Methods, 3*(1), 33-72.
